# Targeting IL-33 in COPD: mechanistic lessons from clinical trials and the path forward

**DOI:** 10.1186/s12931-026-03763-7

**Published:** 2026-06-08

**Authors:** Ivan Tancevski, Thomas Sonnweber

**Affiliations:** Department of Internal Medicine II, Medical University Innsbruck, Innsbruck, Austria

## Abstract

**Background:**

Interleukin-33 (IL-33) is a promising therapeutic target in chronic obstructive pulmonary disease (COPD). However, recent clinical trial setbacks have highlighted the complexity of its biology. This review examines the functions of IL-33 in inflammation, host defense, and tissue remodeling and discusses the potential benefits and harms of targeting this pathway in patients with COPD.

**Methods:**

We synthesize data from clinical trials of anti-IL-33/ST2 agents and integrate these findings with recent molecular and cellular immunology research to elucidate IL-33’s dual functionality, induced by the reduced IL-33 and oxidized IL-33 isoforms. Additionally, we highlight the impact of modifying factors, including multimorbidity, exogenous exposures, and microbial pathogens, on the IL-33 cascade.

**Results:**

Divergent trial outcomes in patients with COPD treated with anti-IL-33 and anti-ST2 antibodies revealed critical mechanistic insights. Inconsistent efficacy of single-pathway inhibitors likely reflect incomplete targeting of IL-33’s dual functionality. While the IL-33red/ST2 pathway drives type 2 inflammation, it is also essential for neutrophil-mediated antibacterial host defense. In contrast, IL-33ox signals through the RAGE/EGFR axis to promote steroid-resistant epithelial remodelling and mucus hypersecretion, a pathway not addressed by ST2-targeted therapies. Experimental data suggesting a potential increase in infection risk associated with anti-IL-33 therapies further underscore the clinical relevance of these mechanistic considerations.

**Conclusions:**

Effective IL-33-targeted therapy in COPD will require a mechanistically comprehensive strategy with precision medicine approaches that address patient heterogeneity, including infection risk and comorbidity burden, and incorporate assessment of IL-33 redox states, soluble ST2, and soluble RAGE.

**Graphical Abstract:**

**Figure:**
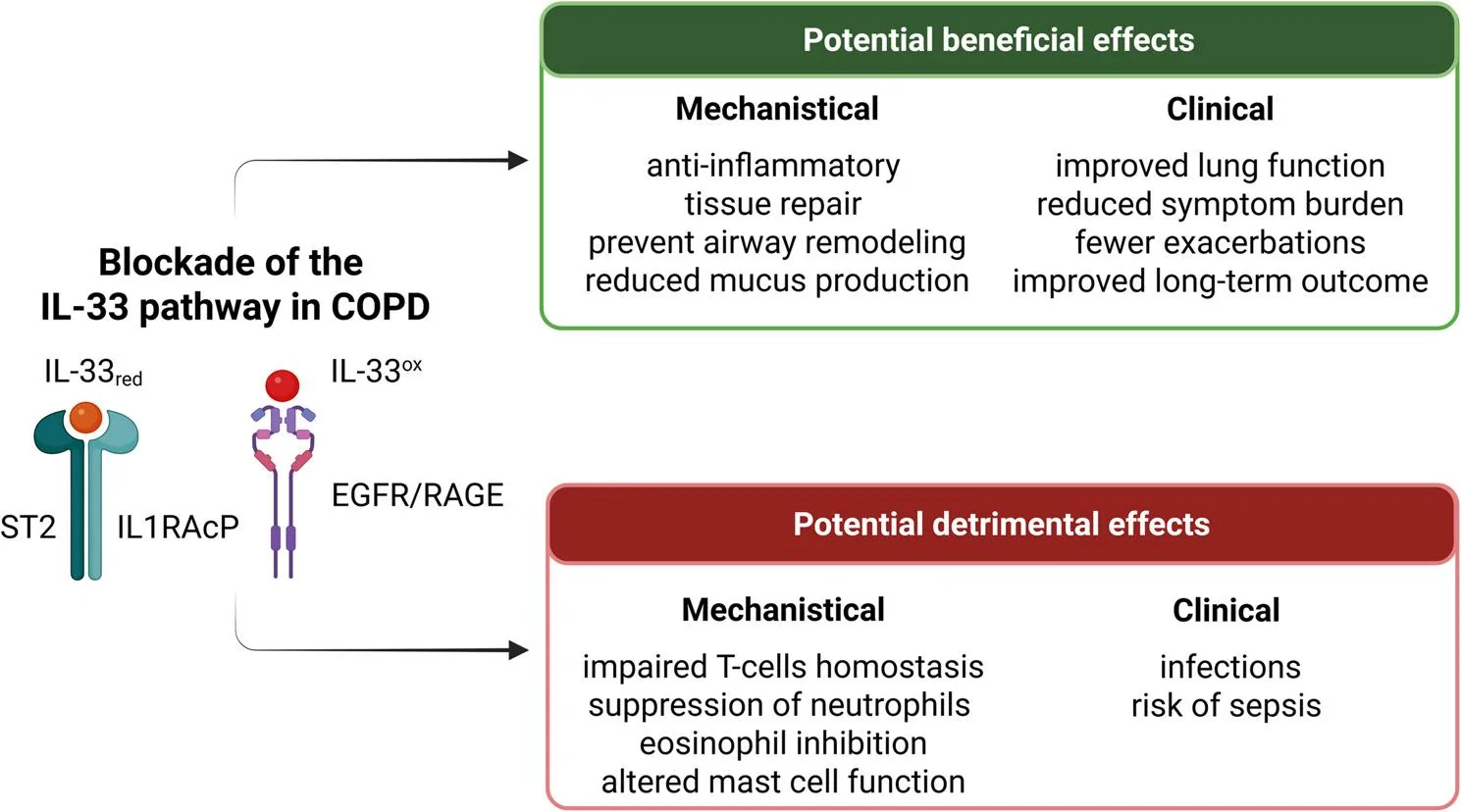
Blockade of the multifunctional IL-33 pathway is a promising therapeutic strategy in chronic obstructive pulmonary disease (COPD). However, owing to the pleiotropic nature of IL-33 signaling, substantial COPD patient heterogeneity, diverse exogenous modifying factors, and variability in treatment strategies, individual therapeutic outcomes may vary considerably.

## Points for clinical practice

- IL-33 has opposing functions in COPD, contributing to pathogenic inflammation, airway remodeling and protective antibacterial host defense.
- The complexity of the IL-33 pathway likely explains inconsistent trial outcomes, as currently tested agents differentially target ST2, IL-33red and IL-33ox/RAGE/EGFR signaling, resulting in differential effects on airway remodeling and neutrophil-mediated bacterial clearance.
- IL-33 targeted therapies should be restricted to biomarker-selected patients, with careful assessment of infection risk and close clinical monitoring, including optimized vaccination strategies.

## Questions for future research

- How can IL-33 pathway activity be precisely stratified in patients with COPD, including reliable clinical assays for IL-33 redox states, sST2, and sRAGE?
- Which COPD endotypes achieve net benefit from IL-33 blockade, balancing anti-inflammatory and anti-remodeling effects against preservation of host defense?
- Can dual-mechanism or combination strategies safely suppress epithelial remodeling while maintaining antibacterial immunity in patients with frequent exacerbations?

## Introduction

Chronic obstructive pulmonary disease (COPD) is characterized by persistent airflow limitation, chronic inflammation, and recurrent exacerbations that drive disease progression and mortality [1]. Despite phenotype-centered treatment algorithms and the recent approvals of dupilumab and mepolizumab for symptomatic COPD patients with elevated eosinophil counts, many patients continue to experience frequent exacerbations, highlighting the need for novel therapeutic approaches [2].

Interleukin-33 (IL-33), an epithelial- and lymphoid cell-derived interleukin-1-like alarmin, has emerged as an attractive therapeutic target [3]. IL-33 is released following tissue damage, infection, or exposure to exogenous inhaled irritants such as pollutants or allergens [4]. Notably, cigarette smoking exerts differential modulatory effects on IL-33 signaling, and a history of smoking is associated with increased IL-33 expression in patients with COPD [5]. IL-33 initiates inflammatory responses that contribute to both protective host defense and pathological tissue remodeling [4]. Three major anti-IL-33 antibodies have advanced to late-stage clinical trials. However, recent setbacks have raised several important questions. A critical advancement has been the recognition that IL-33 exists in two distinct functional states: the reduced form (IL-33red), which signals through the serum stimulation-2 (ST2) receptor to drive type 2 inflammation and neutrophil recruitment for host defense, and the oxidized form (IL-33ox), which engages the receptor for advanced glycation end products (RAGE)/epidermal growth factor receptor (EGFR) signaling complex to promote epithelial remodeling and mucus hypersecretion [6].

This review examines the molecular mechanisms underlying this complex biology and proposes that appreciating IL-33’s roles as a driver of Th2 inflammation and a guardian of antibacterial host defense, as well as its role in tissue remodeling, is essential for understanding recent clinical setbacks and progress in the development of new therapeutic approaches for COPD.

### The complex biology of IL-33: a tale of two pathways

The seemingly contradictory roles of IL-33 can be reconciled by understanding its two major signaling axes, which are determined by its redox state and engagement with distinct receptor complexes, cell types and downstream molecular cascades.

#### The IL-33red/ST2 pathway: bridging type 2 inflammation and host defense

##### Molecular architecture of the canonical IL-33 pathway

**Fig. 1:**
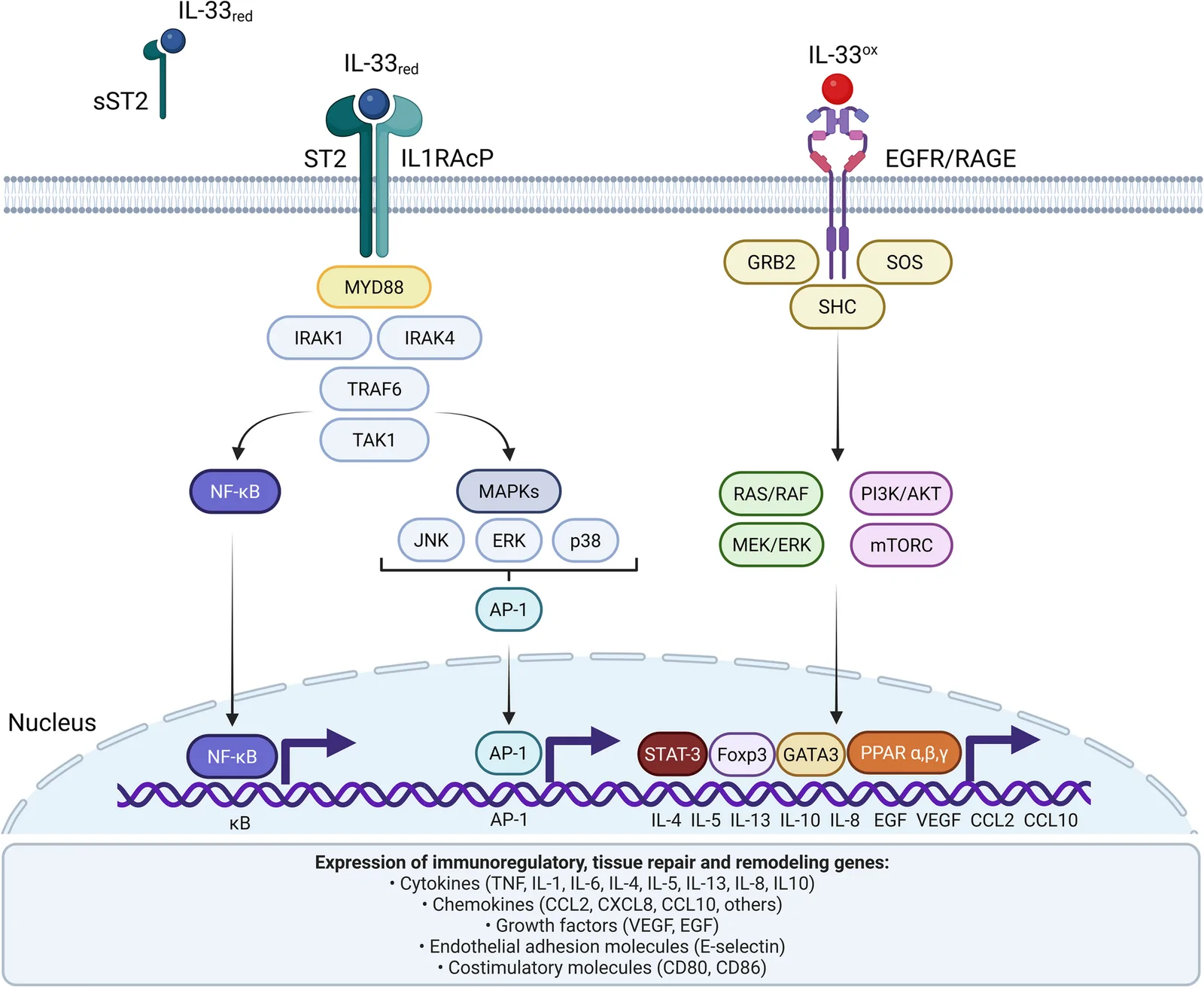
Canonical architecture of the IL-33 signaling pathway. The reduced form of IL-33 (IL-33red) signals through the suppression of tumorigenicity 2/IL-1 receptor accessory protein (ST2/IL1RAcP) complex, which recruits MyD88. The latter engages IL-1 receptor-associated kinases (IRAKs) and activates TNF receptor-associated factor 6 (TRAF6) and transforming growth factor β-activated kinase 1 (TAK1). TAK1 activates and translocates NF-κB, which is related to the key immunomodulatory functions of the IL-33 pathway. Soluble ST2 (sST2) binds free IL-33red and prevents it from binding to the ST2/IL1RAP complex. Additionally, TAK1 induces multiple mitogen-activated protein kinase (MAPK) pathways, contributing to distinct transcriptional pathways involved in immunomodulation and tissue remodeling. The oxidized form of IL-33 (IL-33ox) activates the EGFR/RAGE pathway, which induces the RAS/RAF/MEK/ERK and PI3/AKT pathways and addresses multiple transcription factors, including STAT-3, Foxp3, GATA-3 and PPAR α,β,γ. These factors induce pleiotropic genes, involved in immune surveillance, tissue remodeling, and mucin production

The canonical IL-33 signaling pathway is initiated by the reduced form of IL-33 (IL-33red), which signals through a heterodimeric receptor complex composed of ST2 (IL1RL1) and IL-1 receptor accessory protein (IL1RAcP) [7]. Upon ligand binding, this complex recruits MyD88, which engages IL-1 receptor-associated kinases (IRAKs), specifically IRAK1 and IRAK4. These kinases phosphorylate and activate TRAF6 (TNF receptor-associated factor 6), which, through its E3 ubiquitin ligase function activates the TAK1 (transforming growth factor β-activated kinase 1) complex. TAK1 then phosphorylates and activates the IκB kinase (IKK) complex, leading to NF-κB activation and nuclear translocation. Simultaneously, TAK1 activates multiple mitogen-activated protein kinase (MAPK) pathways, including ERK1/2, JNK, and p38, each of which contributes to distinct transcriptional programs [8]. This proximal signaling module leads to a broad transcriptional response, including pro-inflammatory cytokines, chemokines, and tissue remodeling factors (Fig. 1; Table 1).

**Table 1:** Comparison of the two major IL-33 signaling pathways

Feature	Pathways	
	IL-33red	IL-33ox
Receptor complex	ST2 + IL1RAcP	RAGE + EGFR
Primary ligand	reduced IL-33	oxidized IL-33
Key cells	ILC2s, Th2 cells, mast cells, neutrophils	airway epithelial cells
Primary function	type 2 inflammation and host defense	epithelial remodeling and mucus secretion
Signaling	MyD88/IRAK/TRAF6/NF-κB, MAPKs	EGFR/RAS/RAF/ERK, PI3K/AKT
Transcription factors	NF-κB, AP-1	AP-1, STAT3, Foxp3, GATA3, IRF1, PPAR α, β, γ
Steroid sensitivity	sensitive	insensitive
Pathological outcome	eosinophilia, Th2 inflammation, impaired host defense, oxidative stress	goblet cell metaplasia, mucus hypersecretion, impaired wound healing

The functional diversity of this pathway stems from its activity in numerous immune cells, each expressing ST2 and responding to IL-33 with cell type-specific effector functions. This pleiotropy is both a strength, allowing IL-33 to coordinate complex immune responses, and a challenge for therapeutic targeting, as blocking this pathway simultaneously affects multiple cell types. 

#### Cell-type specific responses and the mast cell amplifier

The ST2 receptor is expressed on a wide array of immune cells, leading to distinct effector functions that collectively orchestrate inflammatory responses. In type 2 innate lymphoid cells (ILC2s), IL-33 is perhaps the most potent activator, inducing the rapid and robust production of IL-5 and IL-13 [9–11]. These cytokines drive eosinophil recruitment, activation, and survival (IL-5), as well as goblet cell metaplasia, mucus hypersecretion, and airway smooth muscle contraction (IL-13). ILC2s, which reside in lung tissue and rapidly respond to epithelial-derived IL-33 without requiring antigen presentation, serve as a critical bridge between innate tissue damage signals and type 2 adaptive immunity. In Th2 cells, IL-33 synergizes with TCR signaling to amplify IL-4, IL-5, and IL-13 production, thereby reinforcing the type 2 inflammatory cascade.

Mast cells are important amplifiers of IL-33 signaling. Upon activation by IL-33, mast cells not only degranulate and release preformed mediators, such as histamine and tryptase, but also produce IL-33, creating a powerful feed-forward amplification loop [12]. A recent study by Alvarado-Vazquez et al. demonstrated that mast cells are required to sustain local IL-33 and CXCL1 levels in the lungs, thereby facilitating the accumulation of T-cells in the bronchoalveolar space in response to IL-33 [12]. Mast cell-derived IL-33 can act in an autocrine manner, further activating mast cells, and in a paracrine manner, recruiting and activating other immune cells. This highlights mast cells as critical amplifiers that bridge the initial alarmin signal to a broader adaptive immune response, potentially explaining why IL-33 responses can be so robust and sustained even after the initial trigger has been resolved.

Eosinophils, while primarily responding to IL-5, also express ST2 and can be directly activated by IL-33, enhancing their survival, degranulation, and production of cysteinyl leukotrienes and other mediators. Basophils respond to IL-33 with enhanced IL-4 production, further skewing the immune response toward a type 2 phenotype. Even regulatory T cells (Tregs) express ST2, and IL-33 can enhance their suppressive function in certain contexts, adding another layer of complexity to the immunomodulatory effects of this pathway.

#### The neutrophil paradox: IL-33 as a guardian of host defense

Although often viewed through the lens of type 2 inflammation, the IL-33red/ST2 axis plays an equally critical and protective role in neutrophil-mediated host defense. This function is paramount in understanding the therapeutic dilemma of IL-33 blockade and represents one of the most clinically relevant aspects of IL-33 biology in COPD. Neutrophils express functional ST2 receptors, and IL-33 signaling is essential for their effective recruitment to sites of bacterial infection. Increased IL-33 expression has been observed in patients with COPD and is correlated with airway and systemic inflammation, including neutrophil activation [13]. Elevated plasma IL-33 levels in patients with COPD are associated with disease severity and exacerbation risk, suggesting that endogenous IL-33 may represent an adaptive response to ongoing tissue damage and infection [14]. The molecular basis for IL-33’s protective role in host defense was elegantly demonstrated by Alves-Filho et al. in a murine sepsis model using cecal ligation and puncture (CLP), a clinically relevant model of polymicrobial abdominal sepsis [15]. They found that IL-33 treatment dramatically improved survival (from ~ 20% to ~ 70%) by enhancing neutrophil influx into the peritoneal cavity and improving bacterial clearance. Conversely, mice lacking ST2 (ST2-/-) showed impaired neutrophil recruitment, higher bacterial loads, and increased mortality rates. The mechanism centres on the chemokine receptor CXCR2, which is essential for neutrophil migration in response to CXC chemokines, such as CXCL1, CXCL2, and CXCL8 (IL-8 in humans).

To understand how IL-33 counteracts threats, it is crucial to examine the mechanisms that impair neutrophil function during infection. During bacterial infection, Toll-like receptor 4 (TLR4) activation by lipopolysaccharide (LPS) from gram-negative bacteria triggers a paradoxical impairment of neutrophil function. TLR4 signaling upregulates G protein-coupled receptor kinase-2 (GRK2) in neutrophils via ERK MAPK pathway activation. GRK2 is a serine/threonine kinase that phosphorylates the C-terminal tail of CXCR2, marking it for β-arrestin-mediated internalization and degradation. This results in a dramatic reduction in surface CXCR2 expression, rendering neutrophils unable to sense and migrate toward chemokine gradients. This state can be described as “neutrophil paralysis”. This phenomenon is thought to represent an endogenous negative feedback mechanism that prevents excessive neutrophil accumulation and tissue damage [16]. However, in the context of severe infection, it becomes maladaptive and impairs the bacterial clearance.

IL-33 counteracts this detrimental effect by inhibiting GRK2 expression at the transcriptional level, thereby preserving surface CXCR2 and maintaining the neutrophil’s ability to migrate [15]. In vitro, IL-33 treatment of LPS-stimulated neutrophils prevented CXCR2 downregulation and restored chemotaxis toward CXCL2. In vivo, the adoptive transfer of neutrophils pre-treated with IL-33 into septic mice resulted in improved bacterial clearance and survival, demonstrating that the effect is cell-intrinsic to neutrophils. This protective effect was validated in humans, where high levels of soluble ST2 (sST2), a decoy receptor that neutralizes IL-33, were associated with increased mortality in patients with sepsis, implying that endogenous IL-33 signaling is crucial for recovery from severe infections [15]. Patients with high sST2 levels exhibited impaired neutrophil CXCR2 expression and reduced chemotactic capacity, mechanistically linking sST2 levels to clinical outcomes.

#### Universal host defense mechanism of the IL-33 pathway

The universality of this mechanism is underscored by the finding that both gram-negative and gram-positive bacterial products converge on the same GRK2/CXCR2 axis, making IL-33’s protective role relevant to the full spectrum of bacterial pathogens encountered in COPD. The same research group demonstrated that TLR2 activation by lipoteichoic acid (LTA) from gram-positive bacteria similarly upregulates GRK2 thereby downregulating CXCR2, impairing neutrophil migration and chemotaxis [17]. In a polymicrobial sepsis model, LTA-treated neutrophils displayed markedly reduced chemotaxis toward CXCL2 and impaired actin polymerization, which is a critical step in cell migration. When adoptively transferred into naïve recipients, these LTA-treated neutrophils exhibit significantly reduced migratory capacity to the site of infection, resulting in impaired bacterial clearance [17].

Remarkably, TLR2-deficient mice showed improved bacterial clearance and survival in polymicrobial sepsis, highlighting the paradoxical detrimental role of excessive pattern recognition receptor activation in neutrophils [17]. This finding underscores that the problem is not a lack of pathogen recognition but rather an overly robust negative feedback response that cripples neutrophil migration. This mechanism is identical to that triggered by TLR4, as previously described. These findings demonstrate that GRK2-mediated impairment of neutrophil migration represents a common pathological endpoint triggered by diverse microbial signals, whether from gram-positive or gram-negative bacteria.

The therapeutic relevance of preserving CXCR2 expression in neutrophils during infection was further validated by pharmacological studies demonstrating that fenofibrate treatment improves survival in sepsis. Our laboratory has shown that this lipid-lowering drug blocks ERK-mediated GRK2 upregulation, thereby maintaining CXCR2 expression and neutrophil chemotactic capacity [18]. In a *Salmonella typhimurium* sepsis model, fenofibrate-treated mice showed dramatically improved survival (from ~ 30% to ~ 80%), which was mechanistically linked to preserved CXCR2 expression on neutrophils, enhanced neutrophil influx to the infection site, and rapid bacterial clearance [18]. Importantly, pharmacological blockade of CXCR2 with the selective antagonist SB225002 completely abolished the protective effects of fenofibrate, confirming that CXCR2 preservation is the critical mechanism underlying these improved results. This pharmacological proof-of-concept demonstrates that preventing CXCR2 downregulation, the same mechanism by which IL-33 protects against sepsis, translates into improved bacterial clearance and survival, validating this pathway as a bona fide therapeutic target.

#### Clinical implications for COPD: the host defense dilemma

For patients with COPD who face frequent infections with both gram-positive organisms, such as *Streptococcus pneumoniae* and *Staphylococcus aureus*, and gram-negative bacteria, including *Haemophilus influenzae*, *Moraxella catarrhalis*, and *Pseudomonas aeruginosa*, the IL-33/CXCR2 axis may represent an important host defense mechanism [13]. Bacterial infections are frequent triggers of COPD exacerbations, accounting for approximately 20–30% of cases [19]. Both TLR2 and TLR4 activation by these diverse pathogens converge on CXCR2 downregulation, creating a state of impaired neutrophil responsiveness that may compromise bacterial clearance. Experimental models suggest that IL-33 counteracts this effect and support antibacterial neutrophil responses across different respiratory pathogens.

These observations raise the hypothesis that blockade of IL-33 signaling could influence antibacterial immunity in COPD. While attenuation of IL-33-driven inflammation may reduce excessive neutrophilic tissue injury, reduced neutrophil recruitment could theoretically also affect pathogen clearance. This consideration may be particularly relevant in COPD, where susceptibility to respiratory infections is already increased because of impaired mucociliary clearance, structural lung damage, and immunosenescence.

In line with this concept, Xia et al. demonstrated that IL-33 expression is increased in COPD and associated with neutrophilic inflammatory responses, supporting a potential role of IL-33 in chronic airway inflammation and tissue injury [20]. Cazzola et al. further highlighted that IL-33-driven neutrophilic inflammation may contribute to tissue damage through the release of elastases and proteases, suggesting that attenuation of this pathway could potentially reduce inflammatory lung injury [21]. At the same time, however, modulation of the IL-33/ST2 axis may also affect innate immune pathways involved in antibacterial defense. Although these considerations remain largely hypothesis-generating and have not been demonstrated clinically, they underscore the complex and potentially context-dependent role of IL-33 in COPD. Accordingly, Riera-Martínez et al. emphasized the complex and context-dependent role of the IL-33/ST2 pathway in COPD [13], while both Cazzola et al. and Varricchi et al. highlighted the potential value of careful patient stratification and immune monitoring in clinical trials evaluating anti-IL-33 therapies [21, 22].

### The IL-33ox/RAGE/EGFR pathway: the epithelial remodeling axis

#### Redox-dependent functional switch

The discovery that extracellular oxidation fundamentally alters IL-33 function by switching its receptor preference from ST2 to RAGE/EGFR represents a paradigm shift [6]. This redox-dependent functional switch positions IL-33 as a sensor of the local oxidative environment, with profound implications for COPD, a disease that is characterized by intense oxidative stress. The lungs of patients with COPD are characterized by an imbalance between oxidants and antioxidants, which is driven by cigarette smoke, air pollution, and chronic inflammation. Reactive oxygen species (ROS) and reactive nitrogen species (RNS) are generated by activated neutrophils, macrophages, and epithelial cells, creating a highly oxidative microenvironment in the lungs [23].

IL-33 contains multiple cysteine residues that are susceptible to oxidation. When released into this oxidative extracellular milieu, IL-33 undergoes oxidation of critical cysteine residues, forming disulfide bonds that alter its tertiary structure. This oxidized form, IL-33ox, loses its ability to bind ST2 with high affinity but gains the ability to interact with RAGE [6]. The oxidative modification of IL-33 thus represents a molecular mechanism by which the local tissue environment dictates the functional outcome of IL-33 signaling, shifting it from an immune-activating alarmin to a pro-remodeling mediator. Figure 1 shows the canonical IL-33 pathway.

#### RAGE as a multi-ligand DAMP receptor in COPD

RAGE is a multi-ligand pattern recognition receptor belonging to the immunoglobulin superfamily [24] and binds various damage-associated molecular patterns (DAMPs), including advanced glycation end products (AGEs), high-mobility group box 1 (HMGB1), S100 proteins (S100A8/A9, S100B), amyloid-β, and, as recently discovered, IL-33ox [6]. RAGE is constitutively expressed at high levels in the lungs, particularly in alveolar type I pneumocytes and airway epithelial cells, where it is thought to play a role in maintaining alveolar architecture. However, in the context of chronic inflammation and tissue damage, RAGE is a central mediator of pathological signaling [25]. In the airways of patients with COPD, multiple RAGE ligands accumulate, creating a state of chronic and steroid-resistant inflammation [26]. For instance, AGEs accumulate due to oxidative stress and glycation of proteins, HMGB1 is released from necrotic cells and activated macrophages, and S100 proteins are secreted by activated neutrophils and epithelial cells. RAGE is dysregulated in the airway epithelium of patients with COPD, amplifying their response to these ligands. For instance, levels of soluble RAGE (sRAGE), a naturally occurring decoy receptor that lacks the transmembrane and cytoplasmic domains and can sequester RAGE ligands, are often deficient in COPD, removing the natural brake on this pro-inflammatory pathway [27]. Accordingly, low sRAGE levels are associated with more severe lung function impairment in COPD, suggesting that sRAGE deficiency contributes to the pathogenesis of the disease [28, 29].

#### The RAGE/EGFR signaling complex and epithelial pathology in COPD

Strickson et al. revealed that IL-33ox forms a signaling complex with RAGE/EGFR in airway epithelial cells [6]. The IL-33ox/RAGE/EGFR complex is a distinct signaling entity with unique downstream consequences. Upon binding IL-33ox and other ligands, RAGE undergoes conformational changes that facilitate its interaction with EGFR. This interaction leads to EGFR transactivation, that is, phosphorylation and activation of EGFR in the absence of its canonical ligands (EGF and TGF-α). The transactivated EGFR then engages its downstream signaling cascades, including the RAS/RAF/MEK/ERK and PI3K/AKT pathways, which drive cell proliferation, survival, and differentiation.

The functional consequences of this pathway activation directly recapitulate the key features of COPD pathology. In primary human bronchial epithelial cells from patients with COPD, IL-33ox treatment induced goblet cell metaplasia, mucus hypersecretion, and reduced ciliated cells [6]. These changes were accompanied by impaired epithelial wound healing and increased epithelial permeability, reflecting the loss of barrier function. Importantly, these effects were not blocked by corticosteroids, explaining the steroid-resistant nature of epithelial dysfunction in many patients with COPD. The IL-33ox/RAGE/EGFR pathway activates transcriptional programs that are largely independent of the classical glucocorticoid-sensitive NF-κB pathway and instead rely on EGFR-dependent activation of transcription factors, such as AP-1 and STAT3.

Critically, these pathological changes were reversed by an antibody that neutralizes IL-33 and prevents its oxidation (similar to tozorakimab), demonstrating that the IL-33ox/RAGE/EGFR pathway is both necessary and sufficient to drive epithelial changes [6]. Gene signatures derived from IL-33ox-stimulated epithelial cells were significantly enriched in airway epithelial brushings from patients with severe COPD compared to those from healthy controls and patients with mild COPD, confirming the clinical relevance of this pathway [6]. This provides a powerful mechanistic explanation for steroid-resistant epithelial dysfunction, hypersecretion, and airway remodeling observed in many patients with COPD, particularly those with frequent exacerbations and rapid disease progression.

#### Steroid resistance and the vicious cycle of oxidative stress

The IL-33ox/RAGE/EGFR pathway may contribute to corticosteroid-insensitive epithelial pathology in COPD. In contrast to the canonical IL-33red/ST2 pathway, which predominantly activates NF-κB-dependent inflammatory signaling and is generally susceptible to glucocorticoid-mediated repression, IL-33ox signals through a ST2-independent RAGE/EGFR complex. This pathway activates EGFR-associated downstream cascades, including ERK-, JNK-, and STAT-related signaling, which may promote epithelial remodeling, mucin hypersecretion, and persistent airway dysfunction despite corticosteroid therapy [6]. EGFR signaling activates transcription factors, such as AP-1 (via ERK) and STAT3 (via JAK), which drive the expression of mucins, inflammatory mediators, and remodeling factors independently of NF-κB. Corticosteroids, which primarily act by inhibiting NF-κB and AP-1 activity through glucocorticoid receptor-mediated repression, exhibit limited efficacy against EGFR-driven transcription.

**Fig. 2:**
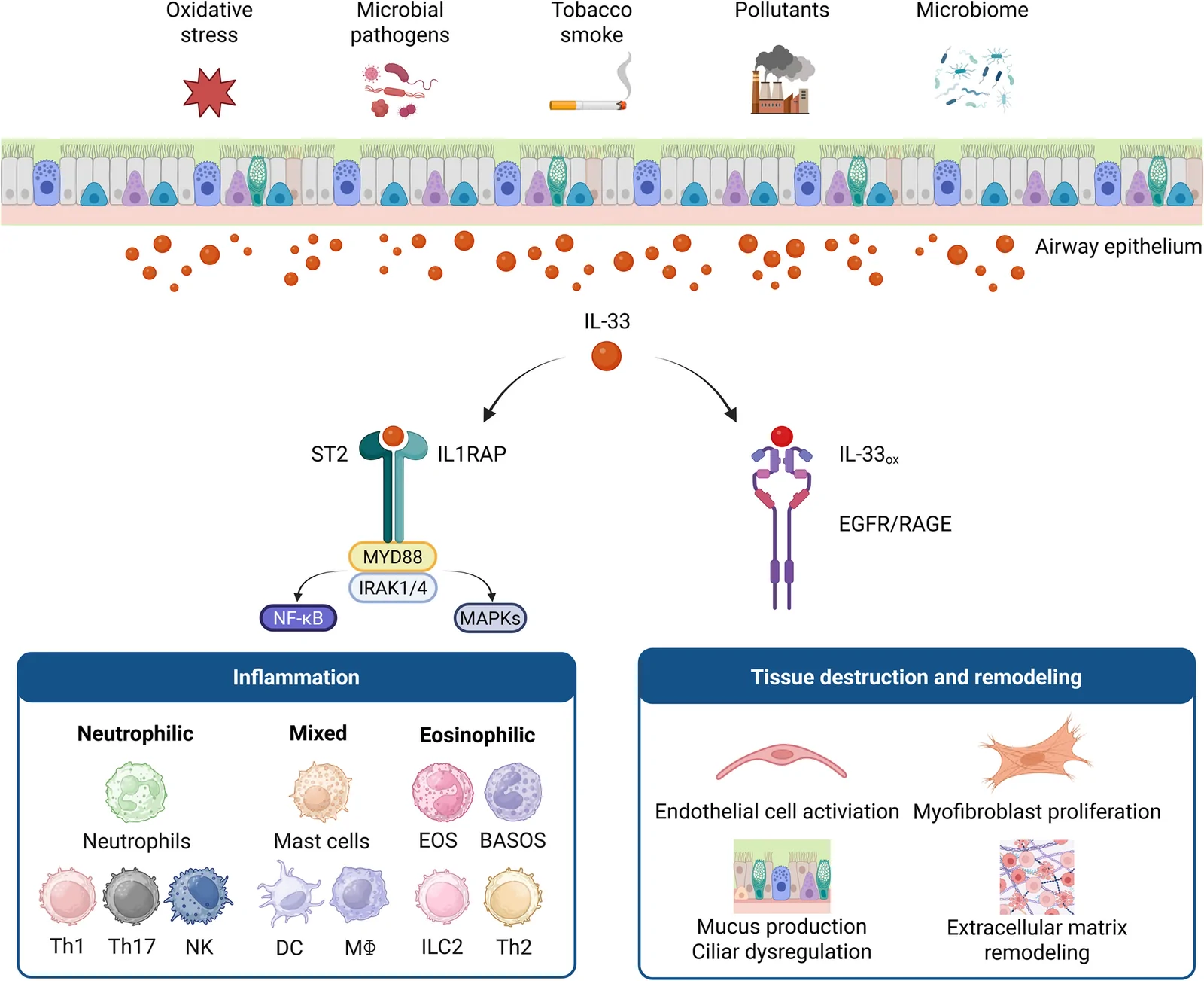
The complex biology of the IL-33 pathway. The complex biological function of the IL-33 pathway derives from its multifaceted signaling pathway, including the IL-33red/ST2 and IL-33ox/RAGE/EGFR axes, and the broad range of cells expressing IL-33 and ST2 receptors, each of which demonstrates cell-type-specific effector functions. Consequently, IL-33 signaling orchestrates complex immune responses and is involved in tissue remodeling and mucus hypersecretion

Furthermore, the oxidative environment in the lungs of patients with COPD creates a vicious cycle. Oxidative stress promotes IL-33 oxidation and generates IL-33ox. IL-33ox, via RAGE/EGFR, induces epithelial dysfunction and inflammation, generating more ROS and RNS from activated immune cells. This perpetuates an oxidative environment, leading to increased IL-33ox formation. Additionally, oxidative stress can directly impair glucocorticoid receptor function by oxidizing critical cysteine residues, further contributing to steroid resistance. This vicious cycle of oxidation, inflammation, and epithelial dysfunction may explain why some patients with COPD show progressive disease despite high-dose inhaled corticosteroid therapy. Figure 2 summarizes the complex biology of the IL-33 pathway.

### Additional layers of regulatory complexity

The IL-33 signaling system is further modulated by multiple regulatory mechanisms, adding layers of complexity and context-dependency to its biological effects.

#### Post-transcriptional regulation by microRNAs

MicroRNAs (miRNAs) are small non-coding RNAs that regulate gene expression post-transcriptionally. Several miRNAs have been identified as regulators of IL-33 expression and signaling. For instance, microRNA-21 (miR-21) is one of the most abundantly expressed miRNAs in the lungs and is generally proinflammatory and profibrotic. miR-21 promotes IL-33 expression in certain contexts, potentially by targeting negative regulators of IL-33 transcription or stabilizing IL-33 mRNA [30]. miR-21 is upregulated in COPD and correlates with disease severity, suggesting that it may contribute to elevated IL-33 levels and sustained inflammation. Therefore, targeting miR-21 could indirectly reduce IL-33 expression and be a therapeutic target for COPD [31]. Additionally, microRNA-328 has also been implicated in the regulation of IL-33-driven inflammatory responses, although its precise mechanism of action is less well-defined [32, 33]. miR-328 may target components of the IL-33 signaling pathway or downstream effector molecules, thereby modulating the intensity and duration of IL-33 responses.

### The influence of multimorbidity and systemic inflammation

COPD rarely occurs in the absence of comorbidities. Comorbidities such as cardiovascular disease (CVD) and type 2 diabetes mellitus (T2DM) are highly prevalent and share inflammatory mechanisms, including neutrophil activation, oxidative stress, and endothelial dysfunction [34]. Notably, patients with COPD have a 2-3-fold increased risk of cardiovascular events and high T2DM prevalence [35, 36]. The shared mechanisms include neutrophil activation and the induction of oxidative stress. In patients with COPD and T2DM, hyperglycemia promotes AGE formation, which are RAGE ligands, potentially amplifying the IL-33ox/RAGE/EGFR pathway. Accordingly, patients with a high comorbidity burden may benefit from therapeutic strategies targeting both the IL-33red and IL-33ox pathways.

### Genetic variation

Genetic variations in the IL-33/ST2 pathway influence disease susceptibility, severity, and therapeutic responsiveness. Single nucleotide polymorphisms (SNPs) in *IL33* and *IL1RL1 (ST2)* genes have been associated with asthma, COPD, and other inflammatory diseases [37].

Several SNPs in *IL1RL1* have been associated with altered expression of soluble and membrane-bound ST2 isoforms, affecting the balance between IL-33 signaling and its inhibition by sST2. Notably, certain *IL1RL1* variants are associated with higher sST2 levels, which could predict reduced responsiveness to anti-IL-33 therapies (as endogenous sST2 neutralizes IL-33). Conversely, variants associated with lower sST2 and higher membrane ST2 expression levels may predict greater benefits from ST2 blockade. *IL1RL1* polymorphisms have also been associated with COPD risk, suggesting that genetic variations in this pathway have clinically meaningful effects [38].

Polymorphisms in the *AGER* gene, which encodes RAGE, have been associated with altered RAGE expression and soluble RAGE (sRAGE) levels. Certain variants are associated with higher RAGE expression and lower sRAGE levels, potentially predisposing individuals to more active RAGE signaling and greater IL-33ox-driven epithelial dysfunction. These patients may be particularly good candidates for dual-mechanism anti-IL-33 antibody therapy [39].

Finally, genetic variations in genes encoding antioxidant enzymes (e.g. *NQO1*, *SOD2*, *and GPX1*) could influence the local oxidative environment in the lungs and the propensity for IL-33 oxidation. Patients with genetic variants associated with reduced antioxidant capacity may have higher IL-33ox levels and could benefit more from therapies that prevent IL-33 oxidation.

#### The role of environmental factors in triggering the IL-33 cascade

Exogenous factors, particularly air pollutants, are critical triggers of pathological processes in COPD and play a central role in activating the IL-33 cascade [40]. Exposure to environmental pollutants, such as ozone (O₃), particulate matter (PM2.5, PM10), diesel exhaust particles, and nanoparticles, leads to the massive production of reactive oxygen species (ROS) in the airways [41]. This oxidative stress causes direct damage to the bronchial epithelium, resulting in disruption of epithelial barrier function. Mechanistically, ROS damage intercellular junction proteins such as claudins, occludin, and zonula occludens-1 (ZO-1) in tight junctions, as well as E-cadherin in adherens junctions, increasing paracellular permeability and leading to a leaky epithelium [42].

This epithelial damage and the resulting cell death, which occurs in part through a ROS-dependent pathway known as oxeiptosis, causes the release of alarmins, prominently including IL-33. The highly oxidative milieu created by pollutants is not only a trigger for IL-33 release but also directly modifies its structure and function. In this environment, reduced IL-33 (IL-33red) is converted to its oxidized form (IL-33ox). This redox modification functions as a molecular switch that fundamentally alters the cytokine’s signaling pathways, as previously described, and may significantly contribute to the biological function of IL-33 in COPD, including pathological epithelial remodeling, goblet cell hyperplasia, and mucus hypersecretion, which are characteristic of COPD and often exhibit steroid resistance. Furthermore, chronic inflammatory processes induced by environmental factors can promote the transcription and release of the soluble receptor sST2 [40]. sST2 acts as a decoy receptor that neutralizes IL-33red and limits its bioavailability. Elevated sST2 concentrations can thus undermine the protective functions of IL-33, such as maintaining CXCR2 expression on neutrophils for effective bacterial defense, and increase the risk of exacerbations. In summary, environmental factors may trigger a cascade of oxidative stress, epithelial barrier disruption, and differential activation of IL-33 redox isoforms, which directly contribute to the pathological hallmarks of bronchial remodeling and mucus hypersecretion in COPD.

### Clinical lessons from anti-IL-33 and anti-ST2 studies

The clinical trial setbacks of first-generation IL-33-targeting biologics can be reevaluated using this multifaceted framework. The mixed results of these clinical trials appear to stem from a mechanistically differential blockade of the IL-33 pathway, complexity of IL-33 signaling, host defense trade-off, and patient heterogeneity (summarized in Table 2).

**Table 2:** Summary of key clinical trials evaluating IL-33 pathway inhibitors in COPD

Agent	Mechanism of Action	Key Clinical Trials	Primary Population	Key Efficacy Outcomes
Itepekimab	Anti-IL-33 mAb (blocks IL-33red/ST2 pathway)	AERIFY-1 (NCT04701983, Phase III) AERIFY-2 (NCT04751487, Phase III)	Former and current smokers with COPD	Inconsistent: 27% exacerbation reduction in AERIFY-1; 2% reduction in AERIFY-2. Less benefit in current smokers.
Astegolimab	Anti-ST2 mAb (blocks IL-33red/ST2 pathway)	ALIENTO (NCT05037929, Phase IIb) ARNASA (NCT05595642, Phase III)	COPD patients with exacerbation history	Phase IIb showed significant exacerbation reduction. Phase III failed to meet primary endpoint.
Tozorakimab	Anti-IL-33 mAb (dual mechanism: blocks ST2 and RAGE/EGFR pathways)	FRONTIER-4 (NCT04631016, Phase IIa) OBERON (NCT05166889, Phase III) TITANIA (NCT05166902, Phase III) MIRANDA (NCT06040086, Phase III)	COPD patients with exacerbation history	Phase IIa: Improved post-bronchodilator FEV1, especially in frequent exacerbators. Phase III: Significant reduction in moderate-to-severe exacerbations across all eosinophil counts and smoking statuses.

### Incomplete blockade of the IL-33 pathway with itepekimab

Itepekimab is a human IgG4P monoclonal antibody which neutralizes IL-33red [43]. Two replicate Phase III trials (AERIFY-1 (NCT04701983) and AERIFY-2 (NCT04751487)) yielded inconsistent results with 27% versus 2% exacerbation reduction for the 2-weeks dosing interval in the treatment groups compared to the placebo control [44, 45]. Itepekimab does not prevent IL-33 oxidation; thus, released IL-33 can be oxidized to IL-33ox, leaving the pro-remodelling RAGE/EGFR pathway fully active. This may permit ongoing epithelial dysfunction and progression of airway remodeling despite the inhibition of the IL-33red/ST2 axis. Second, by blocking the IL-33red/ST2 pathway, itepekimab compromises crucial neutrophil-mediated host defense mechanisms, potentially increasing susceptibility to respiratory infections. A potential explanation for the AERIFY-1 vs. The AERIFY-2 discrepancy lies in the seasonal timing and impact of shielding measures during the COVID-19 pandemic. The latter reduced COPD exacerbation rates in the AERIFY-2 study, which may have affected the outcome. Notably, smoking status influenced the outcomes of the AERIFY trials, with current smokers experiencing less benefit from itepekimab than former smokers.

### The patient selection challenge with astegolimab treatment

Astegolimab is a human IgG2 mAb that blocks the ST2 receptor, preventing all IL-33red signaling [46]. It showed promise in a Phase IIb trial (ALIENTO, NCT05037929), which demonstrated a significant reduction in annualized COPD exacerbation rates in the treatment group compared to the placebo group, but failed in a Phase III study (ARNASA, NCT05595642) [47]. This failure highlights the critical need for patient stratification. For instance, the ARNASA trial did not select biomarkers reflecting ST2 pathway activity (e.g. sST2 levels, *IL1RL1* polymorphisms) or consider patients` infection risk. Without mechanistic biomarker-based selection, the therapeutic window may be excessively narrow. Similar to itepekimab, astegolimab does not target the IL-33ox/RAGE/EGFR pathway. By blocking ST2, astegolimab prevents IL-33red signaling; however, IL-33ox can still engage RAGE/EGFR, driving epithelial remodeling. Furthermore, complete receptor blockade may induce more profound immunosuppression than ligand neutralization, potentially compromising host defense more substantially. Plasma sST2, which serves as a negative feedback regulator for IL-33red signaling and is a biomarker for IL-33 pathway activity, is also bound by astegolimab [48]. While the functional significance of additional sST2 binding remains to be clarified, plasma sST2 measurements lose their utility as biomarker in the context of astegolimab treatment. Finally, astegolimab acts independently of pulsatile IL-33 expression; thus, it may provide a more consistent blockade of the IL-33 pathway than IL-33 targeted treatments.

#### The dual-mechanism approach with tozorakimab

Tozorakimab is engineered to address both IL-33 signaling arms by neutralizing IL-33 and preventing its oxidation [49]. This dual mechanism blocks both the ST2 and RAGE/EGFR pathways. The phase IIa FRONTIER-4 study (NCT04631016) did not meet its primary endpoint (change in pre-bronchodilator FEV1 at week 12) but showed a positive signal for the secondary endpoint of post-bronchodilator FEV1 improvement at week 12 (increase of 67 mL in the tozorakimab treatment group compared to the placebo group). Additionally, the signals were most pronounced in the subgroup of participants with a higher exacerbation burden (≥ 2 prior exacerbations) [50]. Notably, the enhanced efficacy in patients with a high exacerbation burden, coupled with the improvement in post-bronchodilator FEV1, suggests that complete IL-33 pathway blockade may have a mechanistic impact on airway physiology. The safety profile did not show a clear signal of increased infection risk, although long-term studies are needed to definitively assess this.

Based on the FRONTIER-4 results, AstraZeneca initiated a comprehensive Phase III development program for tozorakimab, the LUNA program, comprising four trials: OBERON (NCT05166889), TITANIA (NCT05166902), MIRANDA (NCT06040086), and PROSPERO (NCT05166915). In March 2026, positive high-level results were announced from the replicate Phase III OBERON and TITANIA trials [51]. Both trials enrolled adults with symptomatic COPD and a history of ≥ 2 moderate or ≥ 1 severe exacerbations in the preceding 12 months, irrespective of blood eosinophil count or smoking status. A total of 2,306 patients were randomised across both trials and received tozorakimab 300 mg subcutaneously once every four weeks or placebo on top of standard-of-care inhaled maintenance therapy over 52 weeks. Tozorakimab demonstrated a statistically significant and clinically meaningful reduction in the annualized rate of moderate-to-severe COPD exacerbations compared with placebo in the primary population of former smokers, as well as in the overall population including current smokers, and across all blood eosinophil counts and all stages of lung function severity.

In April 2026, the pivotal Phase III MIRANDA trial also met its primary endpoint [52]. MIRANDA enrolled 1,454 patients with a similar inclusion profile (≥ 2 moderate or ≥ 1 severe exacerbations in the prior 12 months) but evaluated a more frequent dosing regimen of tozorakimab 300 mg every two weeks over 52 weeks. Consistent with OBERON and TITANIA, MIRANDA demonstrated a statistically significant and clinically meaningful reduction in the annualized rate of moderate-to-severe exacerbations in both the primary population of former smokers and the overall population of former and current smokers, across all eosinophil strata and lung function severity stages. Tozorakimab was generally well tolerated in all three trials, with a favourable safety profile consistent with the earlier FRONTIER-4 study.

**Fig. 3:**
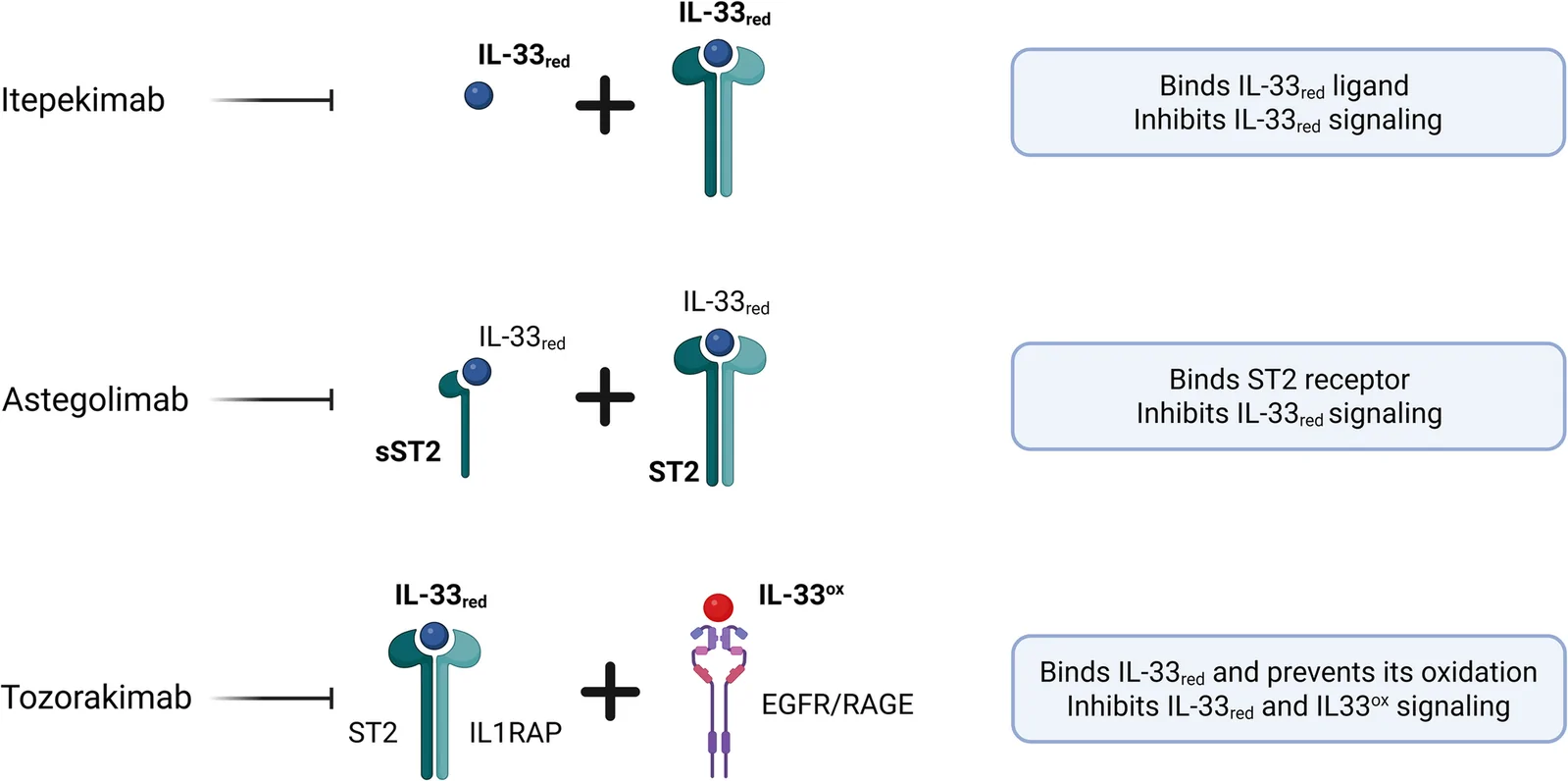
Targeting the IL-33 pathway in clinical trials. Three agents, namely itepekimab, astegolimab, and tozorakimab, have been evaluated in late-stage clinical trials. While itepekimab and astegolimab provide only partial inhibition of the IL-33 pathway by selectively targeting IL-33red signaling and ST-2 signaling, respectively, tozorakimab offers a broader mechanism of action. Specifically, it binds to IL-33red and prevents its oxidation, thus inhibiting both the IL-33red/ST2 pathway and the IL-33ox/RAGE/EGFR signaling axis

The convergent positive results across three independent Phase III trials, enrolling a combined total of more than 3,700 patients, provide robust evidence that dual-mechanism IL-33 blockade delivers clinically meaningful exacerbation reduction in a broad COPD population, independent of eosinophilic phenotype or smoking status. This broad efficacy, which stands in contrast to the eosinophil-dependent benefit observed with dupilumab or mepolizumab, is mechanistically consistent with tozorakimab’s ability to simultaneously neutralize both the IL-33red/ST2 and IL-33ox/RAGE/EGFR pathways, thereby addressing the full spectrum of IL-33-driven pathology. The ongoing PROSPERO extension trial (*n* = 1,713, NCT05742802), which evaluates the annualized rate of only severe exacerbations (hospitalizations and death) over 104 weeks, will provide further insight into the long-term efficacy and safety profile of tozorakimab. Figure 3 provides a summary of currently tested anti-IL-33 therapies.

### The path to precision medicine by biomarker-driven patient stratification

The failures and partial successes of anti-IL-33 therapies underscore the critical need for biomarker-driven patient stratification. Future clinical trials and clinical practice will require sophisticated biomarker strategies to identify patients who are most likely to benefit while minimizing adverse effects, particularly infections.

### IL-33 redox profiling

Assays that can distinguish between IL-33red and IL-33ox in patient samples (serum, sputum, and bronchoalveolar lavage fluid) would be transformative. Patients with predominantly IL-33red expression are predicted to have active ST2-dependent inflammation and may benefit from ST2-targeted therapies, provided that their infection risk is low. Conversely, patients with predominant IL-33ox would be predicted to have active RAGE/EGFR-dependent epithelial remodeling and would likely require dual-mechanism antibodies to achieve benefits.

### RAGE pathway biomarkers

Measuring soluble RAGE (sRAGE) levels may provide insights into RAGE pathway activity. Low sRAGE levels indicate a deficiency in the endogenous decoy receptor, suggesting that RAGE signaling is unopposed and potentially hyperactive. Patients with low sRAGE levels may be particularly good candidates for therapies that block the RAGE pathway.

### Soluble ST2 (sST2) as a biomarker

sST2 is a naturally occurring splice variant of the *IL1RL1* gene that lacks transmembrane and cytoplasmic domains and acts as a decoy receptor that sequesters IL-33 and prevents it from binding to membrane-bound ST2 [15]. Elevated sST2 levels are associated with poor outcomes in multiple diseases. In COPD, high sST2 levels independently predict all-cause mortality, with a hazard ratio of 2.9 [48]. High sST2 levels indicate high endogenous IL-33 activity and suggest that much of the IL-33 has already been neutralized by sST2. Patients with high sST2 levels may benefit from anti-IL-33 therapies but may require higher doses to overcome the high IL-33 burden.

### Gene expression signatures and genetic profiling

Transcriptional profiling can identify gene signatures reflecting IL-33red/ST2 vs. RAGE/EGFR pathway activation. Signatures enriched for Th2 cytokines and ILC2 markers suggest ST2-dependent inflammation, whereas signatures enriched for EGFR target genes and RAGE ligands suggest RAGE/EGFR-dependent remodeling. Genetic variants of *IL1RL1*, *AGER*, and oxidative stress genes could enable genotype-guided therapy selection.

### Infection risk stratification

Given the broad immunological roles of the IL-33 pathway, stratifying patients by infection risk is essential before initiating anti-IL-33 therapy. Risk factors include low FEV1 (< 30% predicted), frequent antibiotic use, history of pneumonia, bronchiectasis, chronic bacterial colonization, and immunosuppressive comorbidities. Patients at high risk of infection may be either excluded from anti-IL-33 trials or subjected to enhanced clinical monitoring.

#### Positioning IL-33 therapies in the clinical algorithm

As the biologic treatment landscape for COPD evolves, positioning IL-33-targeted therapies will require a nuanced clinical algorithm. Currently, dupilumab (anti-IL-4Rα) and mepolizumab (anti IL-5) are approved for COPD patients with an eosinophilic phenotype (typically blood eosinophils ≥ 300 cells/µL) and a history of exacerbations despite LABA/LAMA/ICS therapy. IL-33-targeted therapies, particularly dual-mechanism antibodies like tozorakimab, may occupy a distinct or overlapping niche.

For patients with frequent exacerbations despite maximal inhaled therapy, the initial stratification should rely on blood eosinophil counts. Patients with high eosinophils are prime candidates for dupilumab or mepolizumab. However, for patients with low-to-moderate eosinophils (< 300 cells/µL), or those who fail to respond to dupilumab or mepolizumab, IL-33 blockade may represent a promising alternative, as Phase III data suggest efficacy across all eosinophil strata. Further refinement of this algorithm will likely incorporate novel biomarkers: patients with high sST2 or low sRAGE levels, indicating active IL-33ox/RAGE signaling and epithelial remodeling, may be specifically directed toward dual-mechanism IL-33 inhibitors. Conversely, patients with a high baseline risk of severe bacterial infections (e.g., frequent pneumonias, bronchiectasis) may require cautious evaluation before initiating IL-33 blockade, prioritizing alternative strategies or enhanced monitoring.

### Clinical considerations: the infection risk

Recent reviews have discussed the potential for an increased infection risk with anti-IL-33 therapies [13, 21, 22]. Xia et al. demonstrated that IL-33 expression is increased in COPD and is associated with neutrophil activation, suggesting that endogenous IL-33 may represent an adaptive response to chronic infection and tissue damage [20]. Blocking this adaptive response may leave patients vulnerable to infection. Multiple reviews have emphasized that while the IL-33/ST2 pathway is an attractive therapeutic target, its blockade could weaken innate immunity, which is particularly problematic for patients with COPD who already have an elevated infection risk [13, 21, 22]. Therefore, careful patient selection and monitoring in clinical trials are mandatory.

However, clinical trial data on infection rates are limited. Phase II studies of itepekimab showed no clear increase in infections but were not powered to detect modest increases [44, 45]. Phase III and Phase IV studies with longer follow-up periods are critical. Clinicians should remain vigilant for respiratory infections and pneumonia in patients receiving IL-33 pathway inhibitors. Although current evidence does not demonstrate a clear increase in viral infections associated with IL-33 blockade, preventive strategies such as vaccination against influenza, RSV, pneumococcus, and SARS-CoV-2 remain important in this vulnerable COPD population, particularly given the potential contribution of secondary bacterial infections to exacerbation severity.

## Conclusion

The path forward for IL-33-targeted therapies in COPD is not to abandon the target but to approach it with respect to its complexity. The partial success of clinical trials with itepekimab, astegolimab and tozorakimab have provided invaluable mechanistic insights. IL-33 is not a simple pro-inflammatory cytokine but a multifunctional alarmin with context-dependent roles in inflammation, host defense, and tissue remodeling. Its functional duality, mediated by redox-dependent switching between IL-33red/ST2 and IL-33ox/RAGE/EGFR signaling, creates both therapeutic challenges and opportunities. By embracing mechanistically complete targeting strategies that address both IL-33red and IL-33ox it may be possible to unlock the full therapeutic potential of this pivotal cytokine. However, despite this strong biological rationale, IL-33 targeting may still face significant clinical limitations. The theoretical risk of impaired host defense requires ongoing vigilance, and the profound heterogeneity of COPD patients means that even dual-mechanism therapies will likely require precise, biomarker-driven patient selection to achieve optimal clinical benefit.

## Methods

We performed a systematic literature search using PubMed, Google Scholar, and ClinicalTrials.gov. The authors used the AI tools Manus and Paperpal to enhance the grammar and sentence clarity without contributing to the creation or modification of the scientific content in this manuscript. After using these tools, the authors reviewed and edited the content as needed and take full responsibility for the content of the publication. Figures were created with biorender.com.

## Data Availability

No datasets were generated or analysed during the current study.
